# Goal-Directed Acupuncture in Sports—Placebo or Doping?

**DOI:** 10.1093/ecam/nep210

**Published:** 2011-03-09

**Authors:** Taras I. Usichenko, Vasyl Gizhko, Michael Wendt

**Affiliations:** Department of Anesthesiology and Intensive Care Medicine, Ernst Moritz Arndt University, Friedrich Loeffler Str. 23b, 17487 Greifswald, Germany

## Abstract

The modern pentathlon (MP), sports discipline including fencing, swimming, steeplechase and a cross-country run, requires a rapid change of central nervous and peripheral neuromuscular activity from one sport to another in order to achieve the best possible results. We describe the case where a top MP athlete was supported by a program of acupoint stimulation, which was directed to relieve the symptoms, preventing him from effective performance. Although the fact of acupoint stimulation was associated with improvement of his results, other factors like training effect, placebo and nonspecific physiological effects and their mechanisms in sports are discussed in a literature review. The popularity of complementary and alternative medicine methods among the athletes raises the question of their potential misuse as a doping in competitive sports.

## 1. Introduction

The modern pentathlon (MP), an olympic sports discipline since 1912, includes épée fencing, pistol shooting, 200-m freestyle swimming, a steeplechase and a 3000-m cross-country run. Beyond speed, strength, concentration and intensive endurance performance, the two-day MP contest requires a rapid change of different types of central nervous and peripheral neuromuscular activity in various muscle groups. This is a challenge for athletes to avoid carry-over fatigue from one sport to another in order to achieve the best possible results [1].

## 2. Case Report

We describe the case where a top MP athlete was supported by a program of acupoint stimulation over 7 years of his career. The program of acupoint stimulation was started when the 20-year-old athlete was nominated as a candidate for the national MP team.

At that time the athlete reported that several factors limited his effective sports performance. These factors were severe epigastric pain and knee weakness during running, soreness in the wrist and fatigue of the dominant arm during fencing; general excitement and tremor of the dominant arm during the shooting contest; stiffness of the shoulder muscles during swimming.

The program of acupuncture point stimulation was elaborated according to the symptoms, which have been associated with lower level of performance in each MP event. The decision for the choice of acupuncture points was strengthened by diagnostics, using distant computed scanning thermography [2]. This diagnostic procedure was performed using the “Agema Thermovision 870" device with a measurement resolution of 0.13°C. The primary data, registered by scanning thermography were analyzed using the software, which correlated the localization of the skin areas, where the local temperature was changed, with the anatomical description of acupuncture points [3]. Thermography of the athlete's body was performed at least three times daily after major competitions in which the athlete did not achieve the expected results, his performance being associated with the symptoms described above.

**Figure 1:**
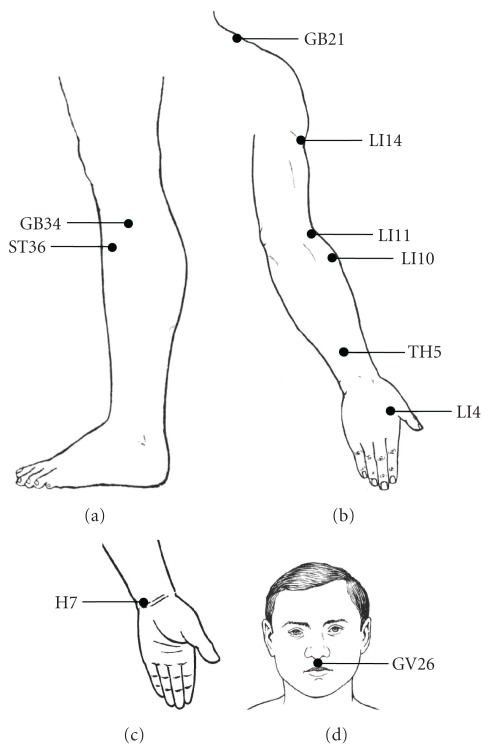
Acupuncture points, used for stimulation to improve the sports' performance in top modern pentathlon athlete. (a) Acupuncture points ST36 and GB34, situated on the proximal latero-anterior surface of the lower leg, were stimulated before running. (b) Acupuncture points of the upper extremities LI4, 10, 11; TH 5 and GB21 were used to relieve arm fatigue during fencing and stiffness of the shoulder muscles during swimming. (c) Acupuncture point H7, situated on the distal ulnar part of the forearm was used to treat general excitement and tremor of dominant arm during shooting. (d) Acupuncture point GV26, situated in the midline of the upper lip, was used, as the point for general tonification. For precise anatomical landmarks of acupuncture points please consult [4].

The acupuncture points, which had local temperature differences on the surface of the skin of the athlete identified by means of thermography, belonged to the stomach (ST36 and ST40), gall bladder (GB 31, 32, 34 and 40) and bladder (BL18 and 19) meridians [4]. Based on these findings the acupuncture points ST36 and GB34 were recommended for stimulation before running (Figure 1(a)). Local acupuncture points were chosen to relieve (Figures 1(b) and 1(c)):

- arm fatigue during fencing—LI4, 10, 11; TH 5;
- stiffness of the shoulder muscles during swimming—LI4, 11, 14; TH5; GB21;
- general excitement and tremor of dominant arm during shooting—H7 and LI4.

In addition, before running and swimming, and during the fencing contest GV26 was stimulated, as the point for general tonification (Figure 1(d)). Acupuncture points were eventually stimulated by pressure and by electromagnetic millimeter waves [5]. The time of stimulation ranged from 5 to 30 min, depending on the goal of treatment. After several applications by an experienced acupuncturist, the athlete himself and his coach were taught to perform the treatment. 

Immediately after the beginning of the acupuncture support program, the athlete improved his performance in 3000-m cross-country running from 10 minutes to <9 minutes and 25 seconds, because epigastric pain and knee weakness, which had been the major limiting factors during running were successfully relieved. Other symptoms (arm fatigue during fencing, stiffness of the shoulder muscles during swimming and general excitement and tremor of dominant arm during shooting) could be also successfully treated with the stimulation of acupuncture points.

**Figure 2:**
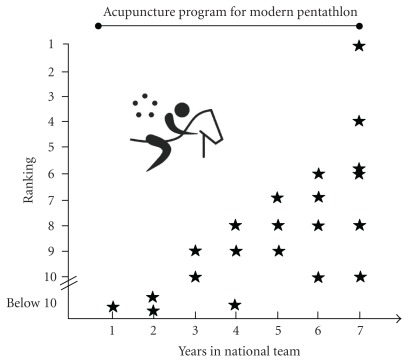
Performance success of the athlete through his sports' career. *x*-axis means the years of sports' performance in national team, *y*-axis means the ranking at the international competitions as black stars (World Cup, World Championships, Olympic games).

Figure 2 shows the increased ranking in international competitions (World Cup, World Championships, Olympic games) from the age of 20 years until the end of his sports career. During that time the athlete was among the best in the national MP team.

The increased success of athlete's performance was attributed to the acupuncture program, although his coach reported a strong psychological effect of the treatment. 

### 2.1. Literature Review

Here we present a case report where the stimulation of acupuncture points in a talented young athlete was strongly associated with rapid significant improvement of his performance. This improvement remained stable under the program during his entire sports career. The choice of the acupuncture points for stimulation was directed to relieve the symptoms, which prevented him from achieving the desired results and was based on the experts' recommendations taken from a standard acupuncture textbook [4] and enhanced by the diagnostics using distant computed scan thermography [2].

We cannot rule out that the improvement of the overall performance described in this case report may have been due to the pure effect of training. However, the possible effects of acupuncture therapy deserve to be further explored, since several experienced athletes and coaches whom we contacted hesitate to explain the rapid and afterwards continuous improvement of his time in 3000 m cross-country running from 10 minutes to <9 minutes 25 seconds as being due to training effect alone. This improvement was achieved mainly due to complete relief from epigastric pain, which had been the main burden to the athlete during previous competitions. The relief other symptoms, which were considered to be the limiting factors (arm fatigue during fencing, stiffness of the shoulder muscles during swimming and general excitement and tremor of dominant arm during shooting) enabled the stable performance in these kinds of sports.

It is known that a single stimulation of acupuncture points in trained athletes may substantially improve their results in light athletics, swimming and cycling. Kaada reported a mean improvement of 2.3 seconds in 800 m track racing (*n* = 5) and 4.3 seconds in 1000 m road racing (*n* = 9) after electric stimulation of LI4 acupuncture point compared to placebo stimulation in a crossover investigation in competitive track-and-field athletes [6].

Regarding the fact that the site-specific acupuncture is effective only for a few conditions in clinical medicine [7, 8] it is plausible to explain the observed effects by non-specific physiological and psychological effects of acupuncture. Especially the placebo effect might play a great role, since placebo, in addition to physiological effects, may constitute 30–50% of the entire clinical effect of acupuncture [8–10].

On the other hand, competitive athletes are extraordinary sensitive to placebo effects. Thus a survey among 48 top professional athletes showed that the majority of them (97%) believe that the placebo effect influences the success of sports performance. Seventy-three percent had experienced a placebo effect during their career and 10 athletes (33%) in the study offered explanations of the nature of the placebo effect [11]. The expectancy-based placebo effect has been shown to produce the same performance improvement in trained athletes, as could be achieved using various pharmacological agents—caffeine and sodium bicarbonate in cyclists [12, 13] and anabolic steroids in weight lifting [14, 15], thus even challenging the specific effect of these drugs.

The enhanced motivation might be the other potential psychological mechanism, resembling the reward framework of placebo pre-conditioning and even acting through the dopaminergic system of basal ganglia, considered now as one of the main mechanisms of placebo [16, 17].

However, there are several reports on specific effects of transcutaneous electric acupuncture point stimulation (TEAS). In a series of experimental crossover investigations in competitive athletes, Kaada has shown that the runners (800 and 1000 m races) and swimmers (100, 200 and 400 m swimming) improved their personal results after TEAS in comparison with sham procedure where sub threshold electric stimuli were applied to acupuncture point LI4 [6]. The cause of the increased physical endurance in athletes in this investigation was suggested to be the result of reduced muscular tension, increased capacity of oxygen transport to the working muscles, increased capacity of the muscles to utilize oxygen and increased muscular microcirculation since the potential psychological factors (like placebo and motivation) were excluded by sufficient blinding of the study participants. The recent investigation of Lin et al. confirms the hypothesis of improved sports performance due to increased oxygen intake in competitive athletes. The authors found out that stimulation of auricular acupuncture points in male boxing athletes led to the enhanced recovery after exercise oxygen consumption using track treadmill in comparison with control condition [18]. Another investigation, performed by So et al. in a crossover manner in healthy volunteers, demonstrated the site-specific effect of TEAS, where stimulation of specific acupuncture points of the calf enhanced the rate of muscle force recovery in comparison with stimulation of non-acupuncture sites [19]. These investigations encouraged us to report the case of goal-directed acupuncture in the modern pentathlon, in order to propose to verify the suggested effects of acupuncture in appropriate randomized controlled trials.

It is interesting, that sports physicians in China treat at least 70% of top athletes using traditional Chinese medicine including acupuncture, where both athletes and medical doctors believe in the energetic nature of acupuncture meridians [20]. Regarding the increasing popularity of complementary and alternative medicine (CAM) among Western athletes—56% of athletes consume CAM in comparison with 36% of normal population [21], the question concerning the putative doping aspect of acupuncture and other CAM methods might be raised due to the existing criteria of doping definition. So far, acupuncture or other CAM methods are not on the list of substances and methods prohibited by World Anti-Doping Agency (WADA) at all times in and out of competition [22]. According to existing WADA criteria, formally acupuncture has a potential to be included in this list. Indeed, according to the World Anti-Doping Code 2009, a substance or method shall be considered for inclusion on the prohibited list if WADA determines that the substance or method meets any two of the following three criteria: (i) medical or other scientific evidence, pharmacological effect or experience that the substance or method, alone or in combination with other substances or methods, has the potential to enhance or enhances sport performance; (ii) medical or other scientific evidence, pharmacological effect or experience that the use of the substance or method represents an actual or potential health risk to the athlete; (iii) WADA's determination that the use of the substance or method violates the spirit of sport described in the Introduction to the Code [23].

We presume that criterion 2 would be irrelevant for acupuncture since rare serious complications, prospectively monitored in thousands of patients, were mainly due to needling procedure itself [24], which can be prevented using other non-invasive stimulation modalities. Nevertheless, according to existing WADA definition of doping, the combination of criteria 1 and 3 might be relevant for acupuncture and other CAM methods. However, if acupuncture has the potential to enhance or enhances sport performance (criterion 1 is fulfilled), we do not believe that the stimulation of acupuncture points violates the spirit of Olympic movement (criterion 2).

Another target for doping suspect might be the potential analgesic mechanism of acupuncture, which is known to be associated with the activation of endogenous opioid system [25]. Interestingly, placebo-induced analgesia is also mediated through the enhanced neurotransmission of endogenous opioids [26]. Recently it was shown that placebo produces measurable opioid-mediated increase of physical performance. Benedetti et al. [27] demonstrated that application of placebo injection on the day of competition induced an opioid-mediated increase of pain endurance and thus enhanced physical performance in healthy volunteers, who were conditioned with only two injections of morphine (one injection per week) before. This effect could be blocked by the administration of opioid-receptor antagonist naloxone. Alone these morphine-like effects of placebo raised the question whether the application of placebo is ethically acceptable in sports competitions, formally throwing the shadow of suspicion on all forms of mental training, psychological interventions and mind–body CAM techniques, which can enhance sport performance. However, we believe that precisely defined WADA criteria concerning CAM therapies, which can be used to enhance sports performance, will relieve these techniques from suspicion of doping potential in the future.

Acupuncture and other CAM techniques, eventually used to enhance the sports performance, should be clearly distinguished from the methods with doping potential. For this purpose the existing WADA criteria concerning CAM methods should be clearly defined based on the experts opinion, involving clinicians, physiologists and specialists on ethics.

Regarding this case report as the first step in the “ladder" of an evidence-based approach in clinical medicine [28], we suggested the idea of goal-directed stimulation of acupuncture points in athletes. As a logical next step the expected “performance-improving" effects of acupuncture and suggested specificity of acupuncture for this application should be verified using appropriate methodology of randomized controlled trials including the updated expert's guidelines on developing research of complex interventions [29].

## Funding

Institutional sources of the Department of Anesthesiology and Intensive Care Medicine, Ernst Moritz Arndt University of Greifswald.
